# The “2.8 ka BP Cold Event” Indirectly Influenced the Agricultural Exploitation During the Late Zhou Dynasty in the Coastal Areas of the Jianghuai Region

**DOI:** 10.3389/fpls.2022.902534

**Published:** 2022-05-23

**Authors:** Xin Jia, Dongsheng Zhao, Michael J. Storozum, Hongwei Shi, Guozhu Bai, Zhen Liu, Zhujun Hu, Liqiang Sun, Qi Wang, Haiming Li

**Affiliations:** ^1^Jiangsu Center for Collaborative Innovation in Geographical Information Resource Development and Application, Nanjing, China; ^2^Key Laboratory of Virtual Geographic Environment (Ministry of Education of PRC), Nanjing Normal University, Nanjing, China; ^3^School of Geography, Nanjing Normal University, Nanjing, China; ^4^Institute of Environmental Archaeology, Nanjing Normal University, Nanjing, China; ^5^School of History, Nanjing University, Nanjing, China; ^6^School of History, Classics, and Archaeology, Newcastle University, Newcastle upon Tyne, United Kingdom; ^7^College of Humanities & Social Development, Nanjing Agricultural University, Nanjing, China; ^8^Institution of Chinese Agricultural Civilization, Nanjing Agricultural University, Nanjing, China; ^9^Agricultural Archaeology Research Center, Nanjing Agricultural University, Nanjing, China

**Keywords:** archaeobotany, bronze age, wheat, abrupt climate, cultural communication

## Abstract

As a global cooling event, many of the climatic and socio-cultural mechanisms that resulted in changes after the 2. 8 ka BP event remain unclear. In China, this period roughly corresponds with the Zhou Dynasty (1046-212 BC), a critical period when ancient Chinese civilization was experiencing significant cultural and technological changes, including the movement of people to modern-day Jiangsu Province, where they intensively used the natural resources found in this the coastal area. Recent archaeobotanical evidence, and two radiocarbon dates on wheat and foxtail millet, indicate that the Datongpu site, which dates around 2,600 cal a BP, was occupied during this period of transition around the 2.8 ka BP climate event. In total, our investigations recovered 3,399 carbonized seeds from seventy-four flotation samples, of which rice, foxtail millet, broomcorn millet, and wheat seeds where predominant along with 2,296 weed seeds. Additionally, we identified several rice spikelets and wheat rachises. The high number of carbonized rice grains indicates that rice farming was the primary crop in an otherwise mixed rice-dry farming system at Datongpu. In addition, we argue that the “2.8 ka BP cold event” probably influenced population growth and caused food shortages throughout Central China, leading people to migrate southeastward along the Huai River to the coastal areas of Jianghuai Region. We argue that this abrupt shift in the climate indirectly facilitated the exploitation and emergence of large-scale agriculture in this area. Our study provides an example for the indirect impact of climate change in areas with relatively favorable climate conditions.

## Introduction

In the past, changes in climatic regimes have had a profound impact on economic development, the distribution of ethnic groups, social stability, and geopolitics [Intergovernmental Panel on Climate Change (IPCC), 2013; Carleton and Hsiang, 2016; Chen S. et al., 2016; FAO, 2016; Hallegatte et al., 2016; Jia et al., 2017a, 2019; Sellers et al., 2019], as well as human migrations, the emergence of social complexity, and, in China, the patterns of dynastic succession (Weiss and Bradley, 2001; Zhang et al., 2008; Buckley et al., 2010; Pederson et al., 2014; Chen et al., 2015a; Timmermann and Friedrich, 2016; Evans et al., 2018). The “2.8 ka BP cold event” was a rapid global climate cooling event, which likely transformed pre-existing social patterns in a variety of ways (Geel et al., 2004; Sophie, 2006). Some transformations thought to be related to this event include technological innovation, population migration, and novel subsistence strategies. The nomadic population migrated toward the south in the pursuit of better meadows around 2.8 ka BP, which accelerated the formation of the Chinese northern nomadic cultural belt (Zhang et al., 2019). This abrupt climate event also led to an increase in the adoption of wheat as a staple crop in the Central Plains of China (Central and Northern Henan Province, southern Shanxi Province, Southern Hebei Province, and central Shaanxi Province), further promoting population growth and socio-political complexity around 2.8 ka BP (Li et al., 2020). However, the relationship between this climate event and human activities in southern China is less clear, because climate changes do not have a significant or direct impact on human activities in areas with high temperature and large amounts of precipitation. Some scholars have argued that rice's low tolerance to cold temperatures in the lower reaches of the Yangtze River (e.g., Chen et al., 2020; Muhammad et al., 2021) coupled with millet and wheat's higher tolerance to cold temperatures in northern China (e.g., Ji et al., 2021; Xiao et al., 2021), influenced the gradual adoption of millet and wheat in southern diets post 2.8 ka BP.

One way to explore the answer to this question is to use archaeobotany to understand cropping patterns in prehistory, which in many ways act as a bridge between climatic processes and ancient societal changes (Mercuri, 2008; Zeder, 2008; Chen et al., 2015b; Jia et al., 2016; Pokharia et al., 2017; Wang et al., 2021). After crops were first domesticated, people transported them all around the world (Jones et al., 2011). Many of the hypotheses regarding origins and diffusion of domesticated crops put climate change as a crucial role (Dalfes et al., 1997; Bawden and Reycraft, 2002; Staubwasser et al., 2003; Bar-Yosef, 2011; Jia et al., 2016, 2021a; Dong et al., 2019). Technological innovation played an important role in accelerating social transformations and human-environmental interactions during the period between the third and first millennium BCE (Diamond and Bellwood, 2003; Chen et al., 2015b; Dong et al., 2017a).

China has a long Neolithic tradition that is divided along geographic lines: the domestication of millet occurred in Northern China (Zhao, 2004a; Lu et al., 2009; Yang et al., 2012; Zhao et al., 2020) and rice was domesticated in the middle and lower Yangtze area of Southern China (Zhao, 1998; Fuller et al., 2009; Wu et al., 2014b), respectively, and then later diffused around the world. However, many studies focused on the diffusion of millet to the West (Jones et al., 2011; Motuzaite-Matuzeviciute et al., 2013; Miller et al., 2016; Dong et al., 2017b) and rice to the South (Fuller, 2011; Deng et al., 2018; Gao et al., 2020), while the adoption of different agricultural systems is understudied in the areas between these two centers of domestication, particularly in the coastal areas of the Jianghuai Region.

A relatively stable shallow sea environment dominated the coastal area of eastern China due to the continuous rise of sea level from 9 to 7 ka BP. After 7 ka BP, sea level regressed and land gradually expanded and advanced in the direction of the ocean (Zheng et al., 2018). As a result, the flat coastal plain of eastern China gradually came into form around 7–6 ka BP (Ling, 1990; Xue, 2002; Li, 2014). Rice farming was the primary subsistence strategy after 5 ka BP in the coastal areas of Jianghuai Region after the land was exposed, which was verified by rice paddy fields at the Tenghualuo site (Lin and Zhang, 2005; Nanjing Museum, 2014) and the rice remains at the Qingdun (Guo, 2000) and Jiangzhuang sites (Wu et al., 2019). However, the transgression likely prevented large-scale human activities until 3,000 BP in the coastal areas of Jianghuai Region, owing to several marine transgressions which occasionally re-occurred before 3 ka BP (Zhao et al., 1994; Li, 2014). The cropping pattern in this area during this time is unclear due to the lack of written records and archaeobotanical evidence, which restricts our ability to examine the relationship between climate change, sea-level fluctuations, cropping patterns, and human settlement.

**Figure 1:**
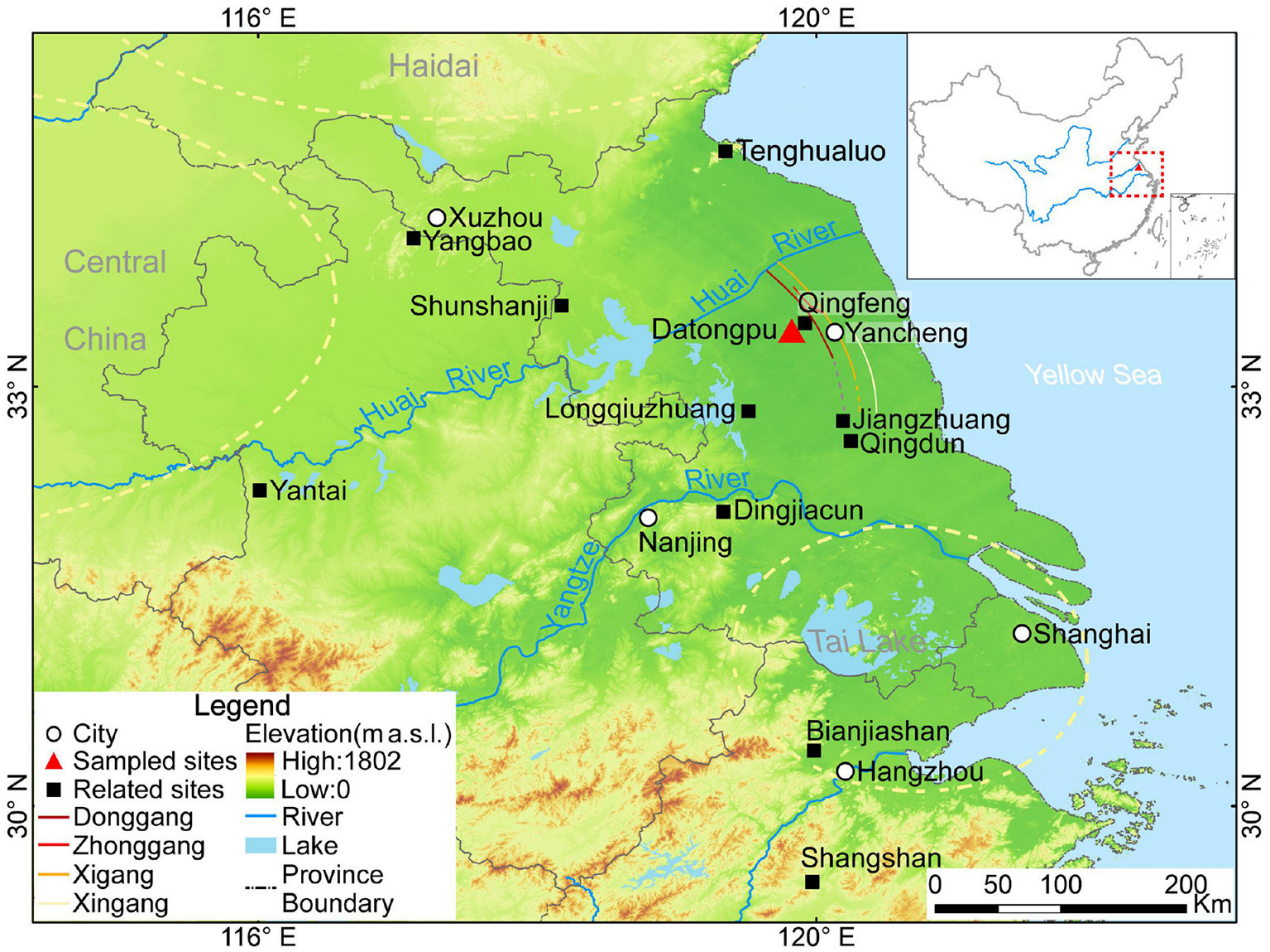
Location of the Datongpu site and related sites in Jiangsu Province and surrounding area (The location of Donggang, Zhonggang, Xigang and Xingang as referred to Zhu et al., 1996).

Nevertheless, new archaeobotanical data from the Datongpu site (Figure 1), enables us to reconstruct the cropping structure during the Zhou Dynasty (1046-221 BC) as the Jianghuai region was reestablished as a coastal area. Combined with paleoclimate records, we are also able to discuss the mechanisms shaping agricultural cropping structures and crop diffusions.

## Study Area and Site Description

The Datongpu site (119.82°E, 33.41°N, 0.1 m a.s.l.) is in Yancheng, Jiangsu Province, in the central coastal area of eastern China and is 73 km away from the coast of the Yellow Sea in the eastern China (Figure 1). There is only about 16 km between the Datongpu site and the ancient coastal sandbars, named Donggang, Zhonggang, Xigang and Xingang (Figure 1) (Zhu et al., 1996). The climate in the coastal areas of Jianghuai Region is in a transitional zone from the subtropical climate in the south to the warm temperate climate in the north, with an average annual precipitation of 785–1,310 mm and a temperature of 13.7–14.5°C (Yancheng Local Chronicles Compilation Committee, 1998). The vegetation type is a mix of deciduous broad-leaf and evergreen broad-leaf vegetation in the north subtropical zone (Yancheng Local Chronicles Compilation Committee, 1998). The main crops include rice, wheat, corn, soybean and rapeseed, and the main animal foods included pigs, sheep, cattle, rabbits, and some aquatic animals (Yancheng Local Chronicles Compilation Committee, 1998).

The coastal areas of the Jianghuai Region are located along the western coast of the Yellow Sea, which are adjacent to the areas between the Haidai Cultural Zone in the north and the Tai Lake cultural zone in the south (Figure 1). The altitude of Central China is higher in the west, and the Huai River was likely a channel of cultural diffusion eastward from Central China. In addition, the flat coastal terrain (1–8.5 m a.s.l.) was likely conducive to the cultural exchange between the north Haidai culture and the south Tai Lake culture. Due to the diverse climatic conditions, dry farming dominated the cropping pattern in Central China and the Haidai zone in the north during the Neolithic, at roughly the same period, rice farming played an important role around the Tai Lake zone in the south (Zhao, 2020).

**Figure 2:**
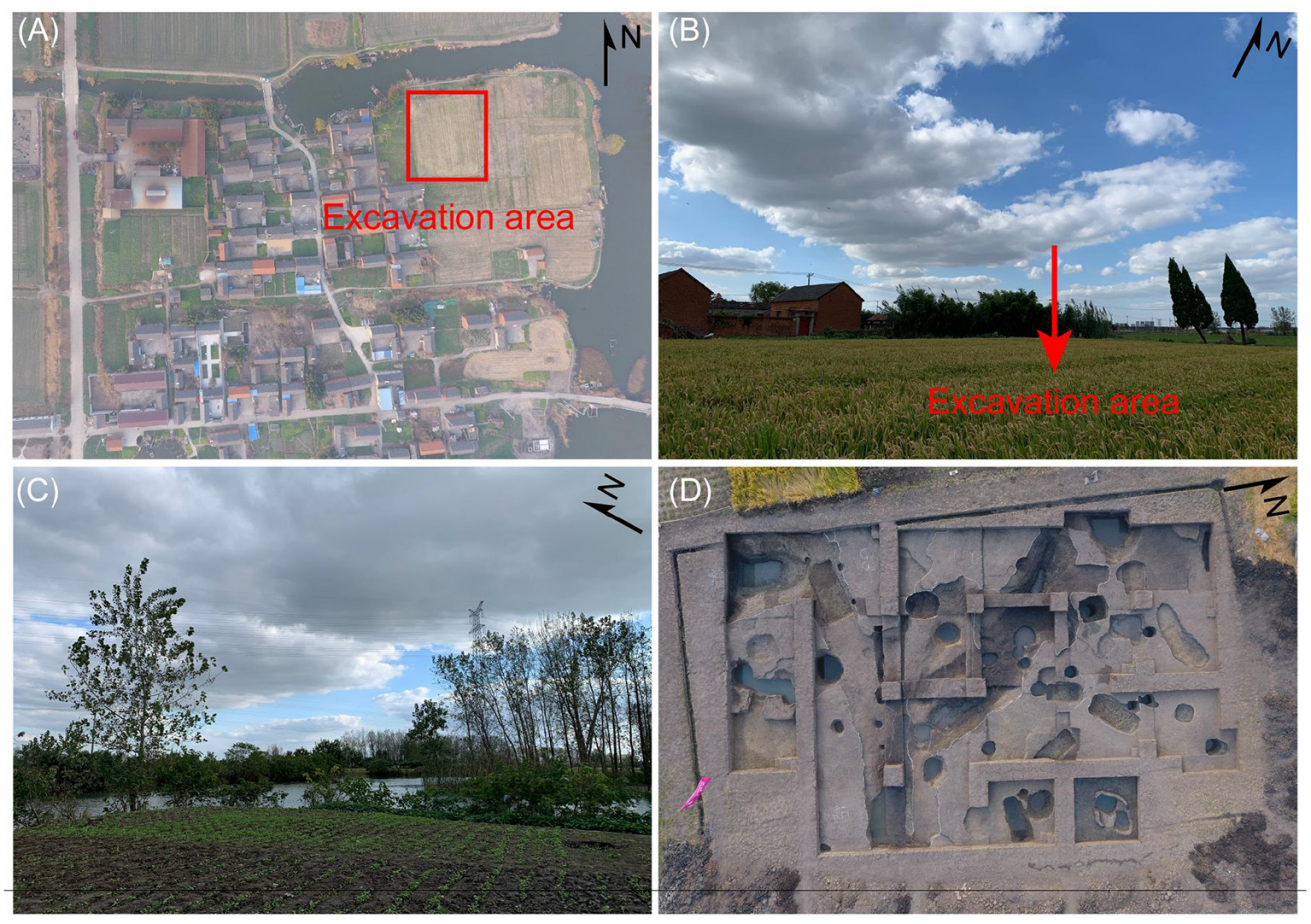
The landscapes of Datongpu site. **(A)** Aerial view of the excavation area of Datongpu site; **(B,C)** Geomorphic map of Datongpu site before excavation; **(D)** Excavation plan of datongpu site.

Datongpu site covers an area of nearly 100,000 m^2^ (Figure 2). In August 2018, the School of History of Nanjing University has started a 5-year long excavation campaign. Many artifacts were recovered through excavation, including pottery, proto-porcelain, bronze, bone, and porcelain. The representative artifacts that date to the Shang and Zhou periods (1600–256 BC) are bronze knives, primitive porcelain cups, pottery *dou* vessel (a type of footed grain serving vessel), and bone hooks. In addition, a large number of animal remains have been found, such as antlers and tortoise shells.

## Methods

A total of 74 soil samples with a volume of 537 liters were collected during the excavation at the Datongpu site in September-December 2019. These soil samples were first placed in buckets, after soaking and cleaning, the light fraction (carbonized plant remains) was collected with a sieve with 80 mesh (aperture size of 0.2 mm) (Zhao, 2004b). Then, the samples were dried in the shade and sorted. The identification on carbonized plant remains was carried out in the Laboratory of Environmental Archaeology, School of Geography, Nanjing Normal University.

The carbonized seeds (wheat from H13 and foxtail millet from H54) were selected for radiocarbon dating with accelerator mass spectrometry (AMS) by Beta Analytic in Miami, Florida, USA. The IntCal20 curve (Reimer et al., 2020) and the Libby half-life of 5,568 years were used to calculate all dates, with the calibration performed using the OxCal 4.4 program (https://c14.arch.ox.ac.uk/oxcal/OxCal.html). All ages reported are relative to AD 1950 (referred to as “cal a BP”).

## Results

### Radiocarbon Dating

Two calibrated ^14^C ages from wheat and foxtail millet collected from the Datongpu site are shown in Table 1. These two calibrated ^14^C ages indicated the age of the site was around 2,600 cal a BP years ago. One calibrated ^14^C age is within the range of 2,738–2,493 cal a BP and belongs to the Chunqiu Period (Early Eastern Zhou Dynasty, 770–476 BC). The other ones are within the range of 2,707–2,365 cal a BP and date to the transition period from the Spring and Autumn Period to the Warring States Period.

**Table 1:** ^14^C dates from the Datongpu site.

**Sample no**.	**Laboratory no**.	**Methods**	**Material**	**^**13**^C/^**12**^C (‰)**	**^**14**^C date (BP)**	**Calibrated age (cal a BP)**	
						**1σ range**	**2σ range**
JS-YC-JH-DTP-H13(3)-W	Beta-566084	AMS	wheat	−23.1	2,460 ± 30	2,699–2,434	2,707–2,365
JS-YC-JH-DTP-H54(2)-F	Beta-566085	AMS	foxtail millet	−9.3	2520 ± 30	2,724–2,518	2,738–2,493

### Flotation

**Figure 3:**
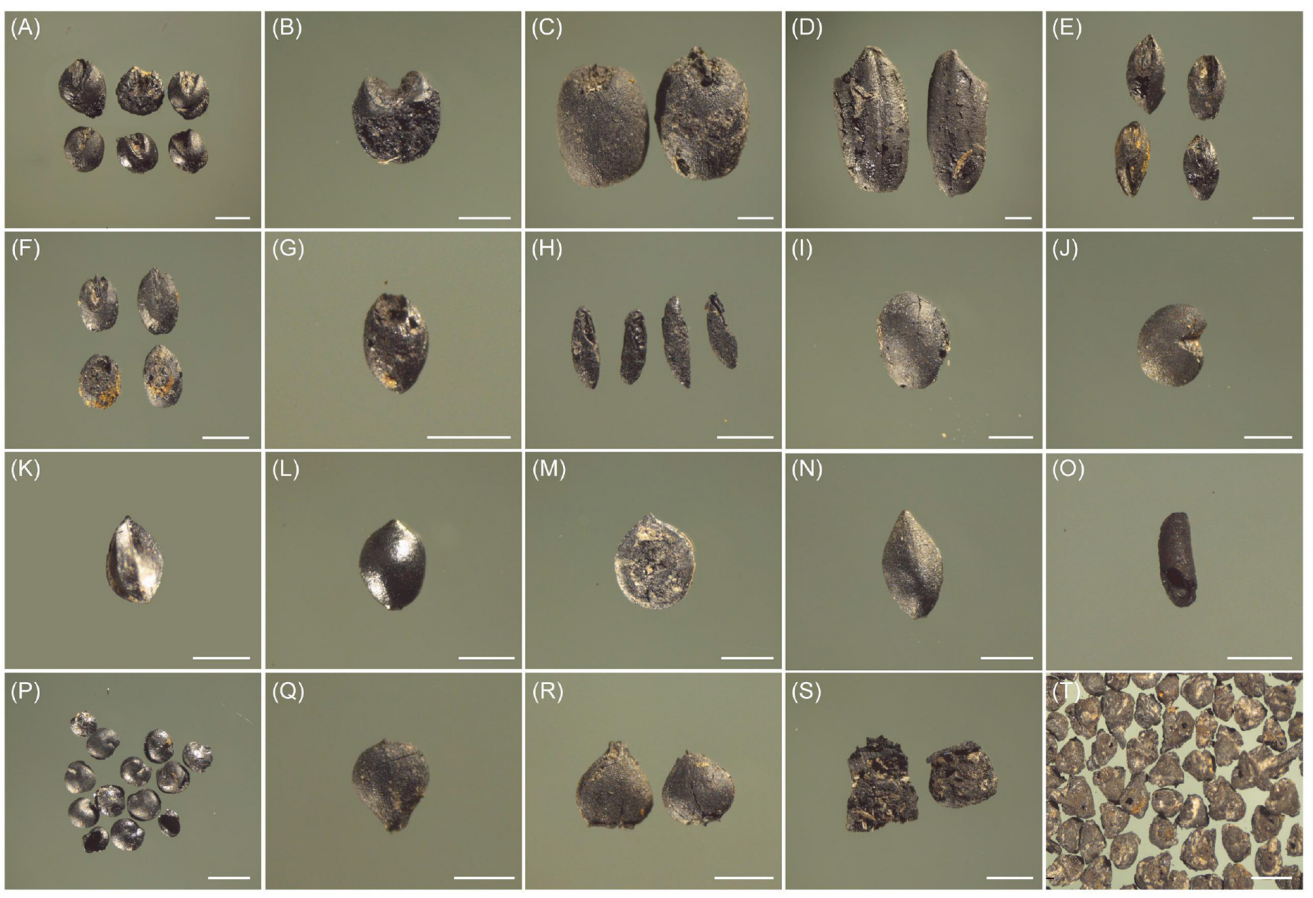
Charred remains collected from the Datongpu site. **(A)** *Setaria italic* **(B)** *Panicum miliaceum* **(C)** *Triticum aestivum* **(D)** *Oryza sativa* **(E)** *Setaria viridi* Beauv. **(F)** *Panicum bisulcatum* Thunb. **(G)** *Echinochloa crusgalli* (Linn.) Beauv. **(H)** *Chloris virgata* Sw. **(I)** *Glycine soja sieb.et* Zucc **(J)** *Astragalus membranaceus* **(K)** *Rumex acetosa* L. **(L)** *Polygonum amphibium* Linn. **(M)** *Polygonum lapathifolium* Linn. **(N)** *Polygonum japonicum* Meisn. **(O)** *Verbena officinalis* L. **(P)** *Chenopodium album* **(Q)** *Scirpus juncoides* Roxb. **(R)** *Carex* Linn. **(S)** Wheat rachis **(T)** Rice spikelet base; scale bar 1 mm.

A total of 5,146 carbonized plant remains were identified from all 74 soils samples, including 3,399 carbonized plant seeds, 1,738 rice spikelet bases and 9 wheat rachises. There are 1,103 crops seeds, which only account for 32.45% of all carbonized seeds, such as foxtail millet (*Setaria italica*), broomcorn millet (*Panicum miliaceum*), wheat (*Triticum aestivum*) and rice (*Oryza sativa*) (Figure 3). Among them, foxtail millet and rice seeds were dominant, followed by wheat and broomcorn millet. Twenty-four types of weed seeds were also found (2,296), including *Setaria viridis* (L.) Beauv., *Echinochloa crusgalli* (Linn.) Beauv., *Chenopodium album* and *Rumex acetosa* L. et al., account for 67.55% of all carbonized seeds. Among them, the seeds of *Rumex acetosa* L. dominated the weed seeds assemblage (74.8%). The quantity of charred seeds collected from the Datongpu site is shown in Table 2. In addition, a total of 52.255 g of carbonized wood (larger than 1 mm) were also floated from the soil samples from the Datongpu site, with an average of 0.97 g/10 L.

**Table 2:** Number of identified carbonized seeds from Datongpu site.

**Plant types**	**Number (grain)**	**Abundance ratio (%)**	**Percentage of crop seeds/weed seeds (%)**	**Ubiquity (%)**
**Crops**	**1,103**	**32.45**	**100.00**	**81.08**
*Setaria italica*	496	14.59	44.97	43.24
*Panicum miliaceum*	6	0.18	0.54	5.41
*Triticum aestivum*	33	0.97	2.99	14.86
*Triticum aestivum frags*	99	2.91	8.98	27.03
Wheat rachis	9	0.26		8.11
*Oryza sativa*	53	1.56	4.81	24.32
*Oryza sativa frags*	416	12.24	37.72	62.16
Rice spikelet base	1,738	51.32		62.16
**Weeds**	**2,296**	**67.55**	**100.00**	**74.32**
*Astragalus membranaceus*	1	0.03	0.04	1.35
*Avena sativa* L.	1	0.03	0.04	1.35
*Carex* Linn.	11	0.32	0.48	5.41
*Chenopodium album*	82	2.41	3.57	17.57
*Chloris virgata* Sw.	9	0.26	0.39	5.41
*Digitaria sanguinalis* (L.) Scop.	2	0.06	0.09	1.35
*Echinochloa crusgalli* (L.) Beauv.	23	0.68	1.00	9.46
*Galium aparine L. var. tenerum* (Gren.et Godr.) Rebb.	2	0.06	0.09	2.70
*Glycine soja* Sieb.et Zucc	18	0.53	0.78	5.41
*Lespedeza bicolor* Turcz	1	0.03	0.04	1.35
*Melibotus albus*	1	0.03	0.04	1.35
*Panicum bisulcatum* Thunb.	16	0.47	0.70	8.11
*Patrinia scabiosaefolia* Fisch	3	0.09	0.13	4.05
*Physali alkekengi* L.	1	0.03	0.04	1.35
*Polygonum japonicum* Meisn.	1	0.03	0.04	1.35
*Polygonum lapathifolium* Linn.	1	0.03	0.04	1.35
*Polygonum amphibium Linn*.	4	0.12	0.17	5.41
*Potamogeton distinctus* A.Bennett	1	0.03	0.04	1.35
*Rumex acetosa* L.	1,719	50.57	74.87	18.92
*Scirpus juncoides* Roxb.	2	0.06	0.09	2.70
*Setaria viridis* (L.) Beauv.	386	11.36	16.81	21.62
*Sporobolus fertilis (Steud.)* W. D. Clayt.	3	0.09	0.13	4.05
*Verbena officinalis* L.	1	0.03	0.04	1.35
*Zizania caduciflora* (Turcz.ex Trin.) Hand. -Mazz	1	0.03	0.04	1.35
Unknown	6	0.18	0.26	5.41
**Total**	**5,146**	100.00		82.43

One thousand seven hundred rumex seeds were collected from one unit of H13, which was likely a dump pit with pieces of broken pottery pieces. The use of rumex may be due to a special type of behavior, which we will study in the future. After excluding the sample of H13, crop seeds account for 64.92% of the total assemblage, and are the dominant part of the macrobotanical assemblage.

## Discussion

### Rice Farming Dominated Subsistence Strategies in the Coastal Area of Jiangsu Province Around 2,600 cal a BP

The proportion and the ubiquity (81.08%) of samples indicates that agriculture dominated the subsistence strategies at the Datongpu site. The 1,103 carbonized grains include foxtail millet (496), broomcorn millet (6), wheat (132) and rice (469). In addition, there were also 1,738 rice spikelet bases and nine wheat rachises. The 469 carbonized rice seeds and 1,738 spikelet bases were identified from 58 samples, and account for 78.38% of all 74 samples. This ubiquity was higher than the ones of foxtail millet (496, 43.24%), wheat (132 seeds, 9 rachises, 35.14%) and broomcorn millet (6, 4.05%). The carbonized plant assemblages demonstrated that rice agriculture dominated the agricultural activities at the Datongpu site, and dry farming also occupied a subordinate position, including foxtail millet, wheat, and broomcorn millet.

Rice farming originated in the middle-lower reaches of the Yangtze River around 10,000 years ago (Zhao, 2011a; Wu et al., 2014b; Zuo et al., 2017) and occupied most of the area south of the Yellow River around 8,500 BP, including the Haidai cultural zone (Crawford et al., 2013; Wu et al., 2013; Jin et al., 2014) and Central China (Zhao and Zhang, 2009; Deng and Gao, 2012; Zhang et al., 2018; Jia et al., 2021b). Rice domestication was also recognized through the identification of starch grains and phytoliths around 8,500 BP at the Shunshanji site in the north area of Jiangsu Province (Zhang et al., 2014; Yang et al., 2016). Afterwards, the earliest carbonized rice remains were identified around 7,000 cal a BP from the Longqiuzhuang site in central Jiangsu Province (Wang and Zhang, 1998; Archaeology Team of Longqiuzhuang Site, 1999). Previous studies considered that rice farming predominated in Jiangsu Province since the Bronze Age (Li et al., 2021) and included the coastal areas of the Jianghuai Region represented by the Datongpu site.

Foxtail millet and broomcorn millet were domesticated in Northern China around 10,000 BP (Lu et al., 2009; Yang et al., 2012; Zhao et al., 2020). Millet agriculture was widespread during the Neolithic to Bronze Age in the Chinese Loess Plateau area (He et al., 2017) and was also scattered throughout Taiwan (Tsang et al., 2017), Fujian (Fu et al., 2016; Zhou et al., 2017; Deng et al., 2018; Dai et al., 2021) and Jiangxi (Chen et al., 2015; Deng et al., 2020). In addition, wheat agriculture was also introduced into Central China at least 3,500 years ago (Zhao, 2011b; Chen, 2016). Dry farming was found at many archaeological sites belonging to the Shang and Zhou dynasties in the upper and middle reaches of Huai River, including Chengyao (Zhong et al., 2018), Guanzhuang (Lan and Chen, 2014), Wangchenggang (Zhao and Fang, 2007), Yangbao (Cheng Z. J. et al., 2016), and Yantai (Anhui Institute of Cultural Heritage Archaeology, 2010). Dry farming was also found at many archaeological sites before 3,000 BP in the middle reaches of Yangtze River, including Xiezidi (Tang et al., 2014), Chengzishan (Tang et al., 2017) and Yejiamiao (Wu et al., 2010). However, evidence of dry farming is rare in the lower reaches of the Huai River (Datongpu site) and Yangtze River (Dingjiacun site, Wu et al., 2017) until 3,000 BP, except for some sporadic evidence from the Shangshan site (Zhao and Jiang, 2016), Bianjiashan site (Zheng, 2014) and Jiangzhuang site (Wu et al., 2019). Therefore, dry farming likely expanded from the west to the east along the Huai River and the Yangtze River basins, which includes the area around the Datongpu site.

In addition, all crop seeds (1,103) accounted for 32.45% of all carbonized seeds (3,399) found at the Datongpu site. If H13 were removed from the total assemblage, due to its position as an outlier with many rumex seeds (1,700), all crop seeds (1,035) accounted for 75.77% of all carbonized seeds (1,366) found at the Datongpu site. The comparison of crops and weeds was used as an indicator of agricultural intensification, the larger quantity of crop seeds, the higher degree of agricultural intensification (Zhao and Xu, 2004). The proportion of crop seeds at the Datongpu site was lower than other sites that date to the Spring and Autumn Period, e.g., Shenmingpu (Liu et al., 2017), Nanwa (Wu et al., 2014a) and Guanzhuang (Lan and Chen, 2014) sites in Henan Province, Zhuguogucheng (Ma et al., 2019) and Kanjiazhai (Chen et al., 2018) sites in Shandong Province. Therefore, the degree of agricultural intensification was relatively low in Datongpu site. Although these Neolithic people engaged in rice-dry farming in Datongpu site, this agricultural structure was likely designed as a response to the specific environment rather than designed as a strategy to obtain as much food as possible.

The plant assemblage found at the Datongpu site indicates that agriculture was dominated by rice farming and supplemented by dry farming around 2,600 cal a BP in the coastal area of Jianghuai Region. Rice farming in this area likely continued from the Neolithic Age in Jiangsu Province, whereas dry farming may have spread along the Huai River from west to east.

### The “2.8 ka Cold BP Event” Indirectly Facilitated Human Exploitation in the Coastal Area of Jiangsu Province

Abrupt climate changes in the past often led to the serious challenges for empires throughout the world (e.g., Weiss and Bradley, 2001; Buckley et al., 2010; Chen et al., 2015a; Jia et al., 2017b; Evans et al., 2018; Xu et al., 2019; Tan et al., 2020a), while favorable climatic conditions promoted the emergence of new civilizations, cultural prosperity and social development again (e.g., Yancheva et al., 2007; Chen et al., 2015a; Putnam et al., 2016; Jia et al., 2017b; Xu et al., 2019). The “2.8 ka cold event” was a strong cold event in the late Holocene (Wanner and Buetikofer, 2008; Vinther et al., 2009; Wang, 2011; Lecavalier et al., 2017; Tan et al., 2020b), which probably corresponded to a significant adjustment in the settlement patterns of human society (e.g., Di Cosmo, 2002; Zhang et al., 2008; Kuzmina, 2015; Li et al., 2020). However, as a pivotal transitional zone between the Haidai cultural zone and the Tai Lake Cultural Zone, the relationship between the “2.8 ka cold event” and human activities in the coastal area of Jianghuai Region, is difficult to define because there are few written records and little archaeological data.

Owing to the relatively stable shallow sea environment which dominated the coastal area of eastern China, the land was submerged during the Holocene Optimum in the eastern coastal area of China (Figure 1), and gradually accumulated sediment and was exposed after 7–6 ka BP (Ling, 1990; Xue, 2002; Li, 2014; Zheng et al., 2018). People began to move eastward to the coastal areas of Jianghuai Region after 5 ka BP (Li et al., 2021). However, owing to the occasional marine transgression, few people settled in this area and instead settled in regions with slightly higher elevations, such as the piedmont area (e.g., Tenghualuo site in Lianyungang City) or knolls (e.g., Qingdun site in Hai'an County) (Li et al., 2021). People engaged in rice farming to ensure the development of civilization after 5 ka BP at the Tenghualuo site (Lin and Zhang, 2005; Nanjing Museum, 2014) and the Qingdun site (Guo, 2000). However, the stratigraphic profiles at the Qingfeng and Qingdun sites indicate that the high sea level decreased around 2.8 ka BP (Zhao et al., 1994; Li, 2014). Further, the Gangxi profile which was 15 km far from Datongpu site, indicates that the sedimentary environment translated to freshwater shallow lake after 2,880 cal a BP, and the Donggang dike developed in the east of Zhonggang (Shu et al., 2021). Therefore, marine transgressions likely prevented large-scale activities until the late Zhou Dynasty in the coastal areas of Jianghuai Region. After that, people may have settled down in the coastal area of the Jianghuai Region without the threat of marine transgression.

**Figure 4:**
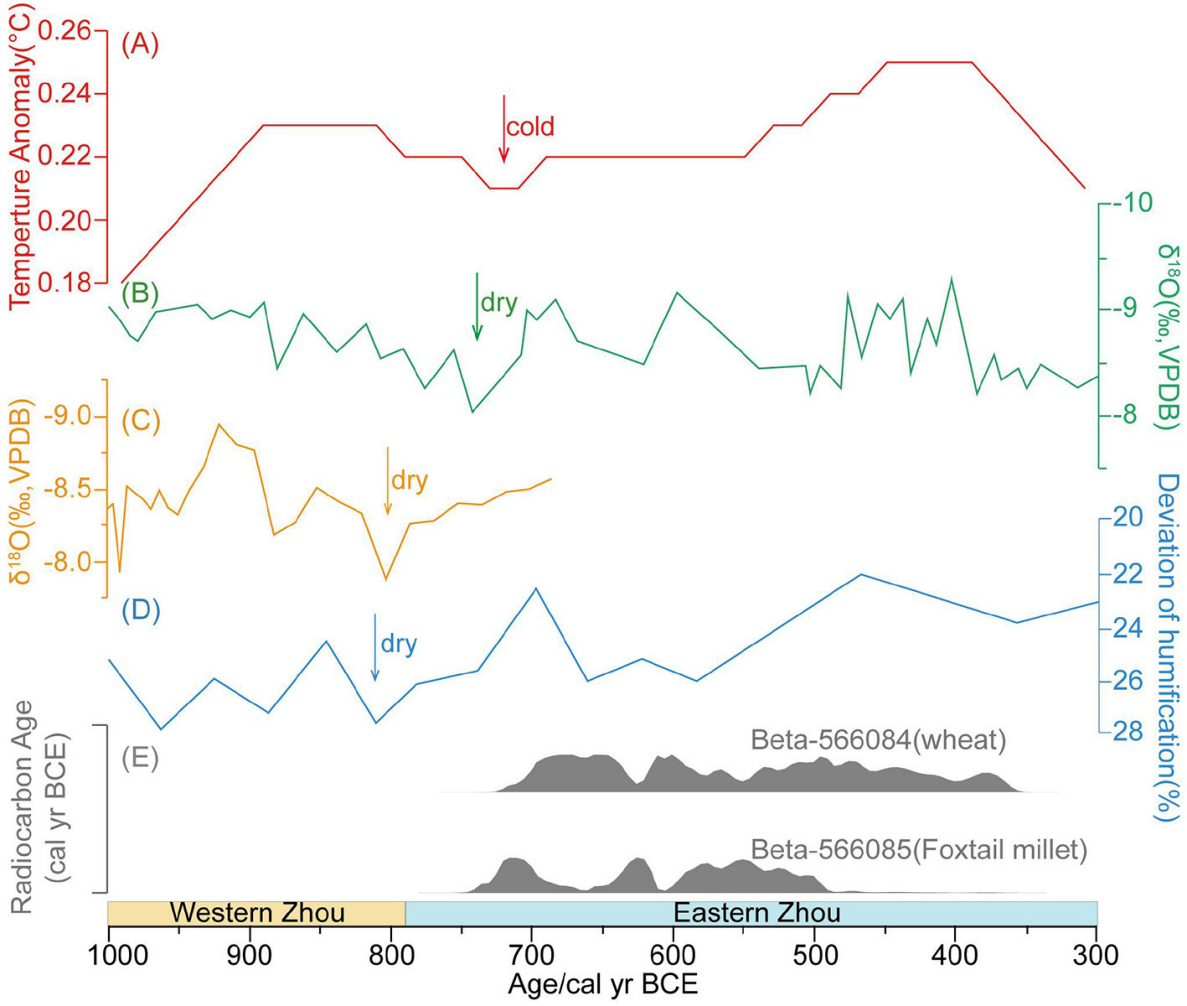
The comparison between climate records and human land use in the coastal area of Jiangsu Province around 2.8 ka BP. **(A)** Temperature anomaly in the Northern Hemisphere (Marcott et al., 2013); **(B)** δ^18^O records from Heshang Cave (Hu et al., 2007); **(C)** δ^18^O records from Wuya Cave (Tan et al., 2020b); **(D)** Record in peat humification in Tianmu mountain Region (Ma et al., 2008); **(E)** Age of Datongpu site (this study).

Climate change had a limited impact on human activities along the coastal areas of Jianghuai Region since the warm and wet climate was favorable for supporting most agricultural activities. Climatic cooling and drought indirectly influenced human activities by sparking migrations from other places, which was a different than the impact that climate changes had on human societies in northern China. A global climate cooling event occurred around 2.8 ka BP (Wanner and Buetikofer, 2008; Vinther et al., 2009; Lecavalier et al., 2017), which was also indicated in the paleoclimatic records in China (Wang et al., 2005; Wang, 2011; Xu et al., 2019; Tan et al., 2020b) (Figure 4). This event was also recorded in a historical document named the “*Zhushu Jinian*” (竹书纪年) which was a chronicle written during the Warring States periods (475-221 BC):

“Frost occurred in July which was the hottest month of the year (779 BC), peach and apricot fruited in October 2 months later than the normal year (772 BC).”

**Figure 5:**
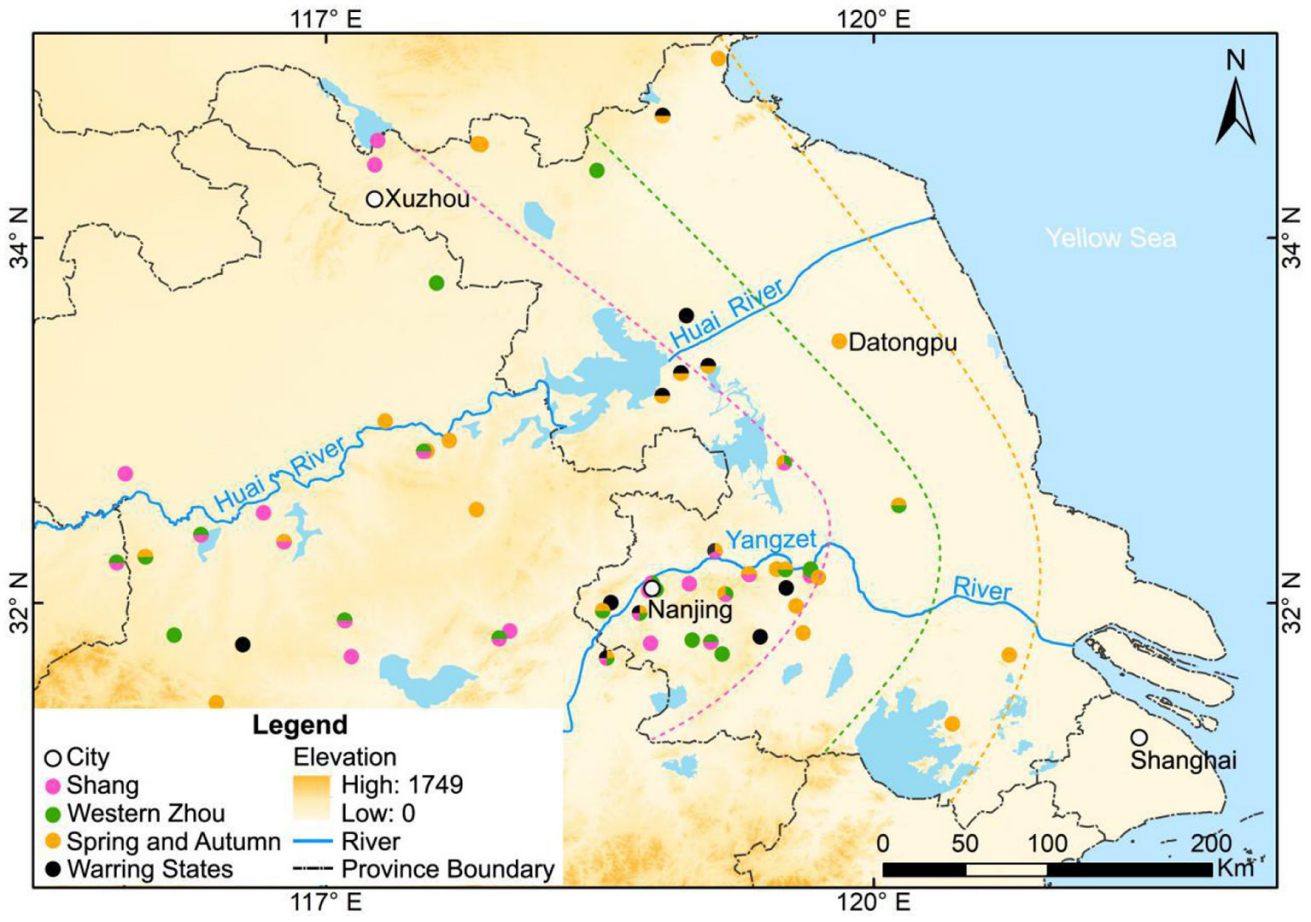
A “waves of advance” diffusion model of the typical artifact “*Li*” from the Central China to the coastal area during the Shang-Zhou Period (The *Li* diffused from the Shang Dynasty to Spring and Autumn Period and probably represented the cultural expansion and human migration from the northwest to the southeast in the Jianghuai Region, which might be related to the coastline and the gradual opening of the coastline for agricultural purposes).

The “2.8 ka BP cold event” likely indirectly facilitated human exploitation in the coastal area of the Jianghuai Region. Some evidence from human bone isotopes indicated that human diets transformed from C4-based foods in 1000-800 BC to C3-based foods during the Eastern Zhou (770–221 BC) in Central China (Li et al., 2020). The transformations of food structure likely promoted grain production (Li et al., 2020), which led to an increased population. Aside from a small number of studies that argue that the population of the Xia and Shang dynasties was over 10 million people (Zhao and Xie, 1988; Colin and Richard, 1992), most studies agree that the population ranged in the millions, rather than the tens of millions (Jiang, 1988; Pang, 1988; Wang, 1990; Song, 1991). The population increased to more than 10 million during the Spring and Autumn Period (770–476 BC) (Pang, 1988; Lu and Teng, 1999; Jiao, 2007) and reached up to more than 30 million people in the Warring States period (476–221 BC) (Zhao and Xie, 1988; Lu and Teng, 1999; Ge, 2002; Jiao, 2007). Given this rapid population increase and the climate event, it is possible that the “2.8 ka BP cold event” may have triggered conflicts and wars more frequently in Central China, leading to food shortages, and likely led to the fall of the Western Zhou Dynasty (1046–771 BC) (Ge, 2010). Therefore, people began to migrate from the Central Plain to the border areas to avoid the disaster of war, seek new resources and exploit new habitats (Wang and Huang, 2002; Li, 2014; Chao, 2018). Sporadic historical data also show that people mainly from Shandong and Henan fled southward through the Jianghuai region in this period. The people of the state of Xu continued to migrate southward because of the expedition of the people of the state of Zhou and may migrated southward through the Jianghuai region and finally reach the present Zhejiang Province (Xu, 2013). Due to the lack of written records from this proto historic period, we must rely on archaeological evidence to explore the potential for cultural diffusion and human migration. *Li* (鬲, an ancient cooking tripod with hollow legs) was a typical pottery vessel used in Central China during the Shang-Zhou Dynasties (Ge and Ge, 2009; The Palace Museum, 2014). The emergence of the *Li* dates to roughly 4,500 years ago in the Yellow River Basin (Zhang, 1997; Shan, 2015). After that, it was brought to many regions with the expansion of culture, until it disappeared during the Warring States Period. The *Li* diffused from the Shang Dynasty to Spring and Autumn Period and probably represented the cultural expansion and human migration from northwest to southeast in the Jianghuai Region (Figure 5). Humans likely moved eastward along the Huai River from Central China to the coastal areas of Jianghuai Region, and dry farming was also brought to supply the rice farming system. Finally, the mixed agricultural pattern appeared widely after 2.8 ka BP in the coastal areas of Jianghuai Region.

Additionally, changes in the East Asian summer monsoon climate caused a decrease from southeast to northwest, and the difference in receiving solar radiation resulted the temperature increased from north to south (Ding, 2021). Therefore, the cold and dry climate may have compelled people to migrate eastward or southward to the comparatively warm and wet conditions in Southern China and Eastern China, which includes the coastal area of Jianghuai Region. As one of the channels for human migration eastward away from the Central Plains, humans likely emigrated eastward along the Huai River from Central China to the coastal areas of the Jianghuai Region, and dry farming was also brought in to supply a rice farming system. Finally, the mixed cropping pattern appeared widely after 2.8 ka BP in the coastal areas of Jianghuai Region.

## Conclusions

The utilization of plant resources was reconstructed in the coastal area of Jianghuai Region based on the charred seeds obtained from Datongpu sites in Yancheng, Jiangsu Province. Rice farming dominated the mixed rice-dry farming around 2,600 cal a BP in the coastal area of Jianghuai Region, and the main crops were rice, wheat, foxtail millet and broomcorn millet.

The “2.8 ka BP cold event” may have intensified the gap between population growth and food storage in Central China, creating a food shortage thus compelling people to migrate eastward or southward to warmer regions. Some people brought dry farming and moved eastward along the Huai River into the coastal areas of Jianghuai Region. The gradual land formation and marine transgression provided a large area of land for human activities after 2.8 ka BP. Their arrival promoted the early exploitation and the emergence of large-scale agriculture and development in the eastern coastal area. Our study provides an example for the indirect impact of climate change in areas with relatively favorable climate conditions.

## Data Availability Statement

The original contributions presented in the study are included in the article/supplementary material, further inquiries can be directed to the corresponding author/s.

## Author Contributions

XJ, DZ, and MS: conceptualization and validation. HS, ZL, LS, and QW: data curation and software. XJ, DZ, MS, GB, ZH, and HL: formal analysis and writing—review and editing. XJ and HL: funding acquisition. XJ, DZ, ZH, and HL: investigation. XJ, DZ, MS, and HL: methodology. XJ and DZ: resources and writing—original draft. XJ: supervision. All authors contributed to the article and approved the submitted version.

## Funding

This research was supported by the National Natural Science Foundation of China under Grant (number 42101152) and the Fundamental Research Funds for the Central Universities under Grant (number SKYC2021011, KYQN2022026).

## Conflict of Interest

The authors declare that the research was conducted in the absence of any commercial or financial relationships that could be construed as a potential conflict of interest.

## Publisher's Note

All claims expressed in this article are solely those of the authors and do not necessarily represent those of their affiliated organizations, or those of the publisher, the editors and the reviewers. Any product that may be evaluated in this article, or claim that may be made by its manufacturer, is not guaranteed or endorsed by the publisher.

## References

[B1] Anhui Institute of Cultural Heritage and Archaeology. (2010). Excavation Report of Zhou Dynasty Settlement in Huoqiu Yantai Huai River Basin. Beijing: Science Press.

[B2] Archaeology Team of Longqiuzhuang Site. (1999). Longqiuzhuang: Excavation Report of Neolithic Sites in Eastern Jianghuai River. Beijing: Science Press.

[B3] Bar-Yosef O. (2011). Climatic fluctuations and early farming in West and East Asia. Curr. Anthropol. 52, S175–S193. 10.1086/659784

[B4] Bawden G. Reycraft R. M. (2002). Environmental disaster and the archaeology of human response. Am. J. Archaeol. 106, 475–476. 10.2307/4126288

[B5] Buckley B. M. Anchukaitis K. J. Penny D. Fletcher R. Cook E. R. Sano M.. (2010). Climate as a contributing factor in the demise of Angkor, Cambodia. Proc. Natl. Acad. Sci. U. S. A. 107, 6748–6752. 10.1073/pnas.0910827107 20351244 PMC2872380

[B6] Carleton T. A. Hsiang S. M. (2016). Social and economic impacts of climate. Science 353, 1112. 10.1126/science.aad9837 27609899

[B7] Chao G. C. (2018). On the influence of climate cooling in the late Shang Dynasty on the replacement of the Shang and Zhou dynasties. Geogr. Teach. Ref. Middle School 12, 66–68.

[B8] Chen F. H. Dong G. H. Zhang D. J. Liu X. Y. An C. B. Ma M. M.. (2015a). Agriculture facilitated permanent human occupation of the Tibetan Plateau after 3600 BP. Science 347, 248–250. 10.1126/science.1259172 25593179

[B9] Chen F. H. Xu Q. H. Chen J. H. Birks H. J. B. Liu J. B. Zhang S. R.. (2015b). East Asian summer monsoon precipitation variability since the last deglaciation. Sci. Rep. 5, 11186. 10.1038/srep11186 26084560 PMC4471663

[B10] Chen S. Chen X. G. Xu J. T. (2016). Impacts of climate change on agriculture: evidence from China. J. Environ. Econ. Manag. 76, 105–124. 10.1016/j.jeem.2015.01.005

[B11] Chen X. X. (2016). Archaeological observation on wheat planting scale in bronze age of China. Agric. His. China 35, 3–9.

[B12] Chen X. X. Ma F. Q. Xu L. G. Bai Y. X. Wang Q. (2018). Flotation results and preliminary analysis of plant remains at site I, area B, qijiazhai site, the ancient city of Qi, Linzi, Shandong. Agric. His. China 37, 15–25.

[B13] Chen X. X. Wang L. C. Niu Z. G. Zhang M. Li C. A. Li J. R. (2020). The effects of projected climate change and extreme climate on maize and rice in the Yangtze River Basin, China. Agr. Forest Meteorol. 282–283, 107867. 10.1016/j.agrformet.2019.107867

[B14] Chen X. X. Zhou G. M. Gong W. (2015). Preliminary analysis of flotation plant remains in Xingan Niucheng, Jiangxi Province from 2006 to 2008. Jianghan Archaeol. 3, 100–108.

[B15] Cheng Z. J. Yang Y. Z. Yuan Z. J. Zhang J. Z. Yu J. Chen B. B.. (2016). Study on carbonized plant remains at yangbao site in Suzhou, Anhui. Jianghan archaeol. 1, 95–103.

[B16] Colin M. Richard J. (1992). Atlas of World Population History. New York, NY: Oriental Publishing House.

[B17] Crawford G. W. Chen X. X. Luan F. S. Wang J. H. (2013). Preliminary analysis of plant remains at the Yuezhuang site in Changqing, Jinan. Jianghan Archaeol. 2, 107–116.

[B18] Dai J. Q. Cai X. P. Jin J. H. Ge W. Huang Y. M. Wu W.. (2021). Earliest arrival of millet in the South Chinacoast dating back to 5,500 years ago. J. Archaeol. Sci. 129, 105356. 10.1016/j.jas.2021.105356

[B19] Dalfes H. N. Kukla G. Weiss H. (1997). Third millennium BC climate change and old world collapse. Springer Sci. Business Media 49, 1–14. 10.1007/978-3-642-60616-8

[B20] Deng Z. H. Gao Y. (2012). Analysis of unearthed Plant Remains at Baligang Site, Dengzhou, Henan. Cult. Relics Southern China 1, 156–163.

[B21] Deng Z. H. Hung H. C. Fan X. C. Huang Y. M. Lu H. Y. (2018). The ancient dispersal of millets in southern China: new archaeological evidence. Holocene 28, 34–43. 10.1177/0959683617714603

[B22] Deng Z. H. Yan Z. Yu Z. (2020). Bridging the gap on the southward dispersal route of agriculture in China: new evidences from the Guodishan site, Jiangxi Province. Archaeol. Anthrop. Sci. 12, 1–10. 10.1007/s12520-020-01117-y

[B23] Di Cosmo N. (2002). Ancient China and Its Enemies: The Rise of Nomadic Power in East Asian History. Oxford: Cambridge University Press. 10.1017/CBO9780511511967

[B24] Diamond J. Bellwood P. (2003). Farmers and their languages: the first expansions. Science 300, 597–603. 10.1126/science.1078208 12714734

[B25] Ding Y. H. (2021). China Climate. Beijing: Science Press.

[B26] Dong G. H. Li R. Lu M. X. Zhang D. J. James N. (2019). Evolution of human–environmental interactions in China from the Late Paleolithic to the Bronze Age. Prog. Phys. Geog. 1, 1–18.

[B27] Dong G. H. Liu F. W. Chen F. H. (2017a). Environmental and technological effects on ancient social the Late Paleolithic to the Bronze Age. Prog. Phys. Geog. 1, 1–18.

[B28] Dong G. H. Yang Y. S. Han J. Y. Wang H. Chen F. H. (2017b). Exploring the history of cultural exchange in prehistoric Eurasia from the perspectives of crop diffusion and consumption. Sci. China Earth Sci. 60, 1110–1123. 10.1007/s11430-016-9037-x

[B29] Evans N. P. Bauska T. K. Gázquez-Sánchez F. Brenner M. Curtis J. H. Hodel D. A. (2018). Quantification of drought during the collapse of the classic Maya civilization. Science 361, 498–501. 10.1126/science.aas9871 30072537

[B30] FAO. (2016). State of Food and Agriculture. Rome: Food and Agriculture Organisation of the UN.

[B31] Fu L. Huang Y. M. Yang H. Wang X. T. (2016). 2014 Excavation of the Calabash Mountain Site in Wuyishan city, Fujian Province. Southeast Cult. 2, 19–36.

[B32] Fuller D. Q. (2011). Pathways to Asian civilizations: tracing the origins and spread of rice and rice cultures. Rice 4, 78–92. 10.1007/s12284-011-9078-7

[B33] Fuller D. Q. Qin L. Zheng Y. F. Zhao Z. J. Chen X. G. Hosoya Y. A.. (2009). The domestication process and domestication rate in rice: spikelet bases from the lower Yangtze. Science 323, 1607–1610. 10.1126/science.1166605 19299619

[B34] Gao Y. Dong G. H. Yang X. Y. Chen F. H. (2020). A review on the spread of prehistoric agriculture from southern China to mainland southeast Asia. Sci. China Earth Sci. 63, 615–625. 10.1007/s11430-019-9552-5

[B35] Ge J. X. (2002). Chinese Population History. Shanghai: Fudan University Press.

[B36] Ge Q. S. (2010). Climate Change in China's Past Dynasties. Beijing: Science Press.

[B37] Ge X. Q. Ge J. H. (2009). Li and It's Culture. Shanxi: Sanqin publishing house.

[B38] Geel B. V. Bokovenko N. A. Burova N. D. Chugunov K. V. Dergachev V. A. Dirksen V. G.. (2004). Climate change and the expansion of the Scythian culture after 850 BC: a hypothesis. J. Archaeol. Sci. 31, 1735–1742. 10.1016/j.jas.2004.05.004

[B39] Guo Y. B. (2000). Analysis of neolithic agricultural economy in the Eastern Jianghuai Region. Agric. Hist. China 1, 3–8.

[B40] Hallegatte S. Bangalore M. Bonzanigo L. Fay M. Kane T. Narloch U.. (2016). Shock Waves: Managing the Impacts of Climate Change on Poverty. Washington, DC: World Bank. 10.1596/978-1-4648-0673-5

[B41] He K. Y. Lu H. Y. Zhang J. P. Wang C. P. Huan X. J. (2017). Prehistoric evolution of the dualistic structure mixed rice and millet farming in China. Holocene 27, 1885–1898. 10.1177/0959683617708455

[B42] Hu C. Y. Henderson G. M. Huang J. H. Xie S. C. Sun Y. Johnson K. R. (2007). Quantification of Holocene Asian monsoon rainfall from spatially separated cave records. Earth Planet. Sc. Lett. 266, 221–232. 10.1016/j.epsl.2007.10.015

[B43] Intergovernmental Panel on Climate Change (IPCC). (2013). Climate Change 2013: The Scientific Basis. Contribution of Working Group I to the Fifth Assessment Report of the Intergovernmental Panel on Climate Change. Cambridge: Cambridge University Press.

[B44] Ji S. P. Ren S. L. Li Y. R. Dong J. Y. Wang L. F. Quan Q.. (2021). Diverse responses of spring phenology to preseason drought and warming under different biomes in the north china plain. Sci. Total Environ. 766, 144437. 10.1016/j.scitotenv.2020.144437 33412432

[B45] Jia X. Lee H. F. Cui M. C. Cheng G. Q. Zhao Y. Ding H.. (2019). Differentiations of geographic distribution and subsistence strategies between Tibetan and other major ethnic groups are determined by the physical environment in Hehuang Valley. Sci. China Earth Sci. 62, 412–422. 10.1007/s11430-018-9301-5

[B46] Jia X. Lee H. F. Cui M. C. Liu C. Zeng L. Yue R. P. H.. (2017a). Habitat variability and ethnic diversity in Northern Tibetan plateau. Sci. Rep. 7, 918. 10.1038/s41598-017-01008-8 28428559 PMC5430525

[B47] Jia X. Li H. M. Lee H. F. Liu Z. Lu Y. Hu Z. J.. (2021b). Agricultural adaptations to topography and climate changes in Central China during the mid-late Holocene. Holocene 31, 1705–1715. 10.1177/09596836211033201

[B48] Jia X. Sun Y. G. Wang L. Sun W. F. Zhao Z. J. Lee H. F.. (2016). The transition of human subsistence strategies in relation to climate change during the Bronze period in the West Liao River Basin, Northeast China. Holocene 26, 781–789. 10.1177/0959683615618262

[B49] Jia X. Wang S. Z. Sun Y. G. Li Y. Y. Jiao Y. J. Zhao Z. J.. (2021a). Charcoal evidence for environmental change ca. 3.5 ka and its influence on ancient people in the West Liao River Basin of northeastern China. Quaternary Res. 102, 142–152. 10.1017/qua.2020.120

[B50] Jia X. Yi S. W. Sun Y. G. Wu S. Y. Lee H. F. Wang L.. (2017b). Spatial and temporal variations in prehistoric human settlement and their influencing factors on the south bank of the Xar Maron River, Northeastern China. Front. Earth Sci. 11, 137–147. 10.1007/s11707-016-0572-5

[B51] Jiang T. (1988). Population and History-A Study of China's Traditional Population Structure. Beijing: People's Publishing House.

[B52] Jiao P. M. (2007). Population research in pre Qin period (Ph.D. diss.), Zhengzhou: Zhengzhou University.

[B53] Jin G. Y. Wu W. W. Zhang K. S. Wang Z. B. Wu X. H. (2014). 8000-Year old rice remains from the north edge of the Shandong Highlands, East China. J. Archaeol. Sci. 51, 34–42. 10.1016/j.jas.2013.01.007

[B54] Jones M. K. Hunt H. V. Lightfoot E. Lister D. L. Liu X. Y. Motuzaite-Matuzeviciute G. (2011). Food globalization in prehistory. World Archaeol. 43, 665–675. 10.1080/00438243.2011.624764

[B55] Kuzmina. (2015). The Prehistory of the Silk Road. Beijing: Science Press.

[B56] Lan W. L. Chen C. Y. (2014). Analysis of plant remains in flotation samples from Xingyang Guanzhuang site. East Asia Archaeol. 1, 402–406.

[B57] Lecavalier B. S. Fisher D. A. Milne G. A. Vinther B. M. Tarasov L. Huybrechts P.. (2017). High Arctic Holocene temperature record from the Agassiz ice cap and Greenland ice sheet evolution. Proc. Natl. Acad. Sci. U. S. A. 114, 5952–5957. 10.1073/pnas.1616287114 28512225 PMC5468641

[B58] Li H. M. Liu Z. James N. Li X. S. Hu Z. J. Shi H. W.. (2021). Agricultural processes revealed by the archaeobotanical evidence and its influencing factors in Holocene in Jiangsu Province, eastern China. Front. Earth Sci. 9, 661684. 10.3389/feart.2021.661684

[B59] Li K. F. (2014). Holocene regression records of Taozhuang and Qingdun sites in Northern Jiangsu (Ph.D. diss.), Nanjing: Nanjing University.

[B60] Li X. Zhang S. J. Lu M. X. Qiu M. H. Wen S. Q. Ma M. M. (2020). Dietary shift and social hierarchy from the Proto-Shang to Zhou Dynasty in the Central Plains of China. Environ. Res. Lett. 15, 1–13. 10.1088/1748-9326/ab6783

[B61] Lin L. G. Zhang W. X. (2005). Study on ancient rice at tenghualuo and houdatang Longshan cultural sites in Huang Huai Region. Southeast Cult. 1, 15–19.

[B62] Ling S. (1990). Evolution of paleogeographic environment in Northern Jiangsu Plain since Holocene. Progr. Marine Sci. 4, 20–28.

[B63] Liu H. Song G. D. Gong Y. W. Jiang H. E. Wang C. S. (2017). Preliminary analysis of plant remains unearthed at the Hengpu site in Xichuan, Henan. Huaxia Archaeol. 1, 54–61.

[B64] Lu H. Y. Zhang J. P. Liu K. B. Wu N. Q. Li Y. M. Zhou K. S.. (2009). Earliest domestication of common millet (*Panicum miliaceum*) in East Asia extended to 10000 years ago. Proc. Natl. Acad. Sci. U. S. A. 106, 7367–7372. 10.1073/pnas.0900158106 19383791 PMC2678631

[B65] Lu Y. Teng Z. Z. (1999). General History of Chinese Population. Beijing: Shandong people's publishing house.

[B66] Ma C. M. Zhu C. Zheng C. G. Yin Q. Zhao Z. P. (2008). Climate change since the late glacial period recorded by high-resolution humification degree of mountain peat in eastern China. Sci. China 9, 1078–1091.

[B67] Ma F. Q. Chen X. X. Lu G. Q. Wang Q. (2019). Analysis on the large remains of plants excavated in 2015 at the ruins of the ancient city of Jiaguo in Zoucheng, Shandong – also on people and plants from the perspective of ancient urban management. Southeast Cult. 3, 69–127.

[B68] Marcott S. A. Shakun J. D. Clark P. U. Mix A. C. (2013). A reconstruction of regional and global temperature for the past 11,300 years. Science 39, 1198–1201. 10.1126/science.1228026 23471405

[B69] Mercuri A. M. (2008). Human influence, plant landscape evolution and climate inferences from the archaeobotanical records of the Wadi Teshuinat area (Libyan Sahara). J. Arid Environ. 72, 1950–1967. 10.1016/j.jaridenv.2008.04.008

[B70] Miller N. F. Spengler R. N. Frachetti M. (2016). Millet cultivation across Eurasia: origins, spread, and the influence of seasonal climate. Holocene 26, 1566–1575. 10.1177/0959683616641742

[B71] Motuzaite-Matuzeviciute G. Staff R. A. Hunt H. V. Liu X. Y. Jones M. K. (2013). The early chronology of broomcorn millet (*Panicum miliaceum*) in Europe. Antiquity 87, 1073–1085. 10.1017/S0003598X0004987519657476

[B72] Muhammad I. A. R. Ding C. Q. Li G. H. Syed T. A. Adel H. Muhammad A. B.. (2021). Vulnerability of rice production to temperature extremes during rice reproductive stage in Yangtze River Valley, China. J. King Saud Univ. Sci. 33, 101599. 10.1016/j.jksus.2021.101599

[B73] Nanjing Museum. (2014). Tenghualuo: Archaeological Excavation Report of Neolithic Sites in Lianyungang City. Beijing: Science Press.

[B74] Pang Z. H. (1988). On labor production mode, productivity and population estimation in the Western Zhou Dynasty. J. Tianjin Normal Univ. 5, 41–50.

[B75] Pederson N. D'Amato A. W. Dyer J. M. Foster D. R. Williams J. W. (2014). Climate remains an important driver of post-european vegetation change in the eastern united states. Global Change Biol. 21, 2105–2110. 10.1111/gcb.12779 25477234

[B76] Pokharia A. K. Rajesh A. Shalini S. Sunil B. Jitendra N. Kumaran R. N.. (2017). Altered cropping pattern and cultural continuation with declined prosperity following abrupt and extreme arid event at ~4,200 yrs BP: evidence from an Indus archaeological site Khirsara, Gujarat, western India. PLoS ONE 12, e0185684. 10.1371/journal.pone.0185684 28985232 PMC5630146

[B77] Putnam A. E. Putnam D. E. Andreu-Hayles L. Cook E. R. Palmer J. G. Clark E. H.. (2016). Little Ice Age wetting of interior Asian deserts and the rise of the Mongol Empire. Quaternary Sci. Rev. 131, 33–50. 10.1016/j.quascirev.2015.10.033

[B78] Reimer P. J. Austin W. E. N. Bard E. Bayliss A. Blackwell P. G. Bronk Ramsey C.. (2020). The IntCal20 Northern Hemisphere radiocarbon age calibration curve (0-55 cal kBP). Radiocarbon 62, 725–757. 10.1017/RDC.2020.41

[B79] Sellers S. Ebi K. L. Hess J. (2019). Climate change, human health, and social stability: addressing interlinkages. Environ. Health Persp. 127, 045002. 10.1289/EHP4534 30986089 PMC6785235

[B80] Shan J. X. (2015). Tao Li Pedigree-the research results of cooking utensils on the tip of the tongue. Cultu. Relics Southern China 1, 1–2.

[B81] Shu Q. Zhao Y. F. Hu Z. Yang P. P. Liu Y. Chen Y.. (2021). Multi-proxy reconstruction of the Holocene transition from a transgressive to regressive coastal evolution in the northern Jiangsu Plain, East China. Palaeogeogr. Palaeocl. 572, 110405. 10.1016/j.palaeo.2021.110405

[B82] Song Z. H. (1991). On the population of Xia and Shang Dynasties. Historical Res. 4, 92–106.

[B83] Sophie L. (2006). The emergence of the Scythians: bronze age to iron age in South Siberia. Antiquity 80, 843–859. 10.1017/S0003598X00094461

[B84] Staubwasser M. Sirocko F. Grootes P. M. Segl M. (2003). Climate change at the 4.2 ka BP termination of the Indus valley civilization and Holocene south Asian monsoon variability. Geophys. Res. Lett. 30, 1425. 10.1029/2002GL016822

[B85] Tan L. C. Dong G. H. An Z. S. Edwards R. L. Li H. M. Li D.. (2020a). Megadrought and cultural exchange along the proto-Silk Road. Sci. Bull. 66, 603–611. 10.1016/j.scib.2020.10.011 36654430

[B86] Tan L. C. Li Y. Z. Wang X. Q. Cai Y. J. Lin F. Y. Cheng H.. (2020b). Holocene monsoon change and abrupt events on the western Chinese Loess Plateau as revealed by accurately-dated stalagmites. Geophys. Res. Lett. 47, e2020GL090273. 10.1029/2020GL090273

[B87] Tang L. Y. Luo Y. B. Tao Y. Zhao Z. J. (2014). Study on the remains of carbonized plants at Xiezidi site in Daye City, Hubei Province. Quaternary Res. 34, 97–105.

[B88] Tang L. Y. Luo Y. B. Zhao Z. J. (2017). Study on the remains of carbonized plants at Chengzishan site in Ezhou, Hubei. Jianghan Archaeol. 2, 108–115.

[B89] The Palace Museum. (2014). A Study on the Pedigree of Chinese Tao Li. Beijing: Palace Museum Press.

[B90] Timmermann A. Friedrich T. (2016). Late Pleistocene climate drivers of early human migration. Nature 538, 92–95. 10.1038/nature19365 27654920

[B91] Tsang C. H. Li K. T. Hsu T. F. Tsai Y. C. Fang P. H. Hsing Y. I. C. (2017). Broomcorn and foxtail millet were cultivated in Taiwan about 5000 years ago. Bot. Stud. 58, 1–10. 10.1186/s40529-016-0158-2 28510186 PMC5430587

[B92] Vinther B. M. Buchardt S. L. Clausen H. B. Dahl-Jensen D. Johnsen S. J. Fisher D. A.. (2009). Holocene thinning of the Greenland ice sheet. Nature 461, 385. 10.1038/nature08355 19759618

[B93] Wang C. L. Zhang M. (1998). Restudy on the remains of primitive rice cultivation at Qiuzhuang Site in Gaoyou. Agric. Archaeol. 1, 3–5.

[B94] Wang H. Huang C. C. (2002). Climate change and social change in the middle reaches of the Yellow River at the end of Shang Dynasty. J. Historical Sci. 1, 13–18.

[B95] Wang J. Zhou X. Y. Xu H. Liu J. C. Yang Q. J. Zhao C.. (2021). Relationship between C4 biomass and C4 agriculture during the holocene and its implications for millet domestication in Northeast China. Geophys. Res. Lett. 48, 1–11. 10.1029/2020GL089566

[B96] Wang S. W. (2011). The Holocene Climate Change. Bejing: China Meteorological Press.

[B97] Wang Y. J. Cheng H. Edwards R. L. He Y. Kong X. An Z.. (2005). The Holocene Asia nmonsoon:links to solar change sand North Atlantic climate. Science 308, 854–857. 10.1126/science.1106296 15879216

[B98] Wang Y. M. (1990). On population in pre Qin period. J. Shanghai Normal Univ. 2, 33–42.

[B99] Wanner H. Buetikofer J. (2008). Holocene bond cycles: real or imaginary. Geografie 113, 338–349. 10.37040/geografie2008113040338

[B100] Weiss J. H. Bradley C. R. (2001). what drives societal collapse? Science 291, 609–610. 10.1126/science.1058775 11158667

[B101] Wu C. R. Liu H. Zhao Z. J. (2010). Discussion on prehistoric agriculture in Jianghan Plain from flotation results of Yejiamiao site in Xiaogan. Cult. Relics Southern China 4, 65–69.

[B102] Wu W. W. Lin L. G. Gan H. Y. Yan L. (2019). Settlement production activities in Liangzhu period of Jiangzhuang site from the perspective of plant remains. Agric. His. China 38, 3–16.

[B103] Wu W. W. Si H. W. Wang S. M. Li Y. J. (2017). Preliminary analysis of carbonized plant Remains at Dingjia Village Site, Zhenjiang, Jiangsu. Southeast Cult. 5, 78–88.

[B104] Wu W. W. Zhang J. H. Jin G. Y. (2014a). Plant archaeological evidence from Erlitou of nanwa site in Dengfeng, Henan Province to settlement agriculture in the Han Dynasty. Cult. Relics Central China 1, 109–117.

[B105] Wu W. W. Zhang K. S. Wang Z. B. Jin G. Y. (2013). Analysis of plant Remains of Xihe Site (2008) in Zhangqiu. East Asia Archaeol. 373–390, 477–478.

[B106] Wu Y. Jiang L. P. Zheng Y. F. Wang C. S. Zhao Z. J. (2014b). Morphological trend analysis of rice phytolith during the early Neolithic in the Lower Yangtze. J. Archaeol. Sci. 49, 326–331. 10.1016/j.jas.2014.06.001

[B107] Xiao D. P. Liu D. L. Feng P. Y. Wang B. Cathy W. Shen Y. J.. (2021). Future climate change impacts on grain yield and groundwater use under different cropping systems in the north china plain. Agr. Water Manage. 246, 106685. 10.1016/j.agwat.2020.106685

[B108] Xu D. K. Lu H. Y. Chu G. Q. Liu L. Shen C. M. Li F. J.. (2019). Synchronous 500-year oscillations of monsoon climate and human activity in Northeast Asia. Nat. Commun. 10, 1–10. 10.1038/s41467-019-12138-0 31511523 PMC6739325

[B109] Xu F. (2013). The flow of artifacts and the migration of people during Pre-Qin Period in the Jianghuai area. J. Changjiang Cult. 12, 161–172.

[B110] Xue C. T. (2002). Discussion on the relationship between Holocene sedimentary environment and sea level change in Qingfeng section, Jianhu, Jiangsu. Acta Sedimentol. Sin. 20, 174–177.

[B111] Yancheng Local Chronicles Compilation Committee. (1998). Yancheng City Annals. Nanjing: Jiangsu Science and Technology Press.

[B112] Yancheva G. Nowaczyk N. R. Mingram J. Dulski P. Schettler G. Negendank J. F. W.. (2007). Influence of the intertropical convergence zone on the East Asian monsoon. Nature 445, 76–77. 10.1038/nature05431 17203059

[B113] Yang X. Y. Wan Z. W. Perry L. Lu H. Y. Wang Q. Zhao C. H.. (2012). Early millet use in northern China. Proc. Natl. Acad. Sci. U. S. A. 109, 3726–3730. 10.1073/pnas.1115430109 22355109 PMC3309722

[B114] Yang Y. Z. Cheng Z. J. Li W. Y. Yao L. Li Z. Y. Luo W. H.. (2016). The emergence, development and regional differences of mixed farming of rice and millet in the upper and middle Huai River Valley, China. Sci. China Earth Sci. 46, 1037–1050. 10.1007/s11430-015-5340-3

[B115] Zeder M. A. (2008). Domestication and early agriculture in the Mediterranean Basin: origins, diffusion, and impact. Proc. Natl. Acad. Sci. U. S. A. 105, 11597–11604. 10.1073/pnas.0801317105 18697943 PMC2575338

[B116] Zhang J. Z. Cheng Z. Z. Lan W. L. Yang Y. Z. Luo W. H. Yao L.. (2018). New Progress in plant archaeology research of Jiahu Site in Wuyang, Henan Province. Archaeol. 4, 100–110.

[B117] Zhang J. Z. Li W. Y. Yin C. L. Cheng Z. J. Yang Y. Z. Luo W. H.. (2014). Main achievements of plant remains analysis at shunshanji site in Sihong, Jiangsu. East Asia Archaeol. 1, 365–373.

[B118] Zhang Q. Li X. Z. Wang Q. Ye H. Y. Zhu H. Qin Y. G.. (2019). Osteological evidence of violence during the formation of the Chinese northern nomadic cultural belt in the Bronze Age. Archaeol. Anthrop. Sci. 11, 6689–6704. 10.1007/s12520-019-00934-0

[B119] Zhang Z. P. (1997). The rise of aerial tripods in the Yellow River Basin. Chin. Archaeol. 1, 30–48, 113.

[B120] Zhang Z. P. Cheng H. Edwards R. L. Chen F. H. Wang Y. J. Yang X. Y.. (2008). A test of climate, sun, and culture relationships from an 1810-year Chinese cave record. Science 322, 940–942. 10.1126/science.1163965 18988851

[B121] Zhao W. L. Xie S. J. (1988). Chinese Population History. Beijing: People's Publishing House.

[B122] Zhao X. T. Tang L. Y. Shen C. M. Wang S. H. (1994). Holocene climate change and sea level change in Qingfeng section of Jianhu, Jiangsu Province. Acta Oceanol. Sin. 1, 78–88.

[B123] Zhao Z. J. (1998). The middle Yangtze region in China is one place where rice was domesticated: phytolith evidence from the Diaotonghuan cave, northern Jiangxi. Antiquity 72, 278. 10.1017/S0003598X00087524

[B124] Zhao Z. J. (2004a). The Origin of Dry Farming in north China Was Discussed From the Flotation Results of Xinglonggou Site. East Asian Antiquities (Vol. A). Beijing: Cultural Relics Press.

[B125] Zhao Z. J. (2004b). Flotation: a field technique of Paleothnobotany for recovering plain remains. Archaeology 3, 80–87.

[B126] Zhao Z. J. (2011a). New archaeobotanic data for the study of the origins of agriculture in China. Curr. Anthropol. 52, S295–S305. 10.1086/659308

[B127] Zhao Z. J. (2011b). Characteristics of agricultural economic development during the formation of Chinese civilization. J. Natl. Museum China 1, 19–31.

[B128] Zhao Z. J. (2020). Origin if agriculture and archaeobotabotanical works in china. Agrc. His. China 3, 3–13.

[B129] Zhao Z. J. Fang Y. M. (2007). Flotation results and analysis of Wangchenggang site in Dengfeng. Huaxia Archaeol. 2, 78–89.

[B130] Zhao Z. J. Jiang L. P. (2016). Analysis of the remains of plants unearthed from the flotation of the Shangshan site in Pujiang, Zhejiang. Cult. Relics Southern China 3, 109–116.

[B131] Zhao Z. J. Xu L. G. (2004). The results and preliminary analysis on pilot flotation at wangjiazui of Zhouyuan site. Cult. Relics 10, 89–96.

[B132] Zhao Z. J. Zhang J. Z. (2009). Analysis report of 2001 flotation results of Jiahu Site. Archaeology 8, 84–93.

[B133] Zhao Z. J. Zhao C. H. Yu J. C. Wang T. Cui T. X. Guo J. N. (2020). Results and analysis of plant flotation at donghulin site in Beijing. Archaeology 7, 99–106.

[B134] Zheng H. B. Zhou Y. S. Yang Q. Hu Z. J. Ling G. J. Zhang J. Z.. (2018). Spatial and temporal distribution of Neolithic sites in coastal China: sea level changes, geomorphic, evolution, and human adaption. Sci. China Earth Sci. 61, 123–133. 10.1007/s11430-017-9121-y

[B135] Zheng Y. F. (2014). Analysis of Plant Seed and Fruit Remains. Zhejiang Institute of Cultural Relics and Archaeology: Bianjiashan. Beijing: Cultural Relics Press.

[B136] Zhong H. Zhang Y. Q. Wu Q. Zhao Z. J. (2018). Analysis of floatation results from the Chengyao site of Dengfeng in Henan Province. Agric. Archaeol. 6, 7–16.

[B137] Zhou Z. Y. Huang Y. M. Fan X. C. Fu X. G. Wang X. Y. Wei C. F.. (2017). 2013 Excavation briefing of Nanshan Ruins Cave 4, Mingxi County, Fujian Province. Archaeology 10, 3–22.

[B138] Zhu C. Cheng P. Lu C. C. Wang W. (1996). Analysis of coastline evolution in the Yangtze River Delta and coastal areas of Northern Jiangsu since 7000 BP. Sci. Geogr. Sin. 16, 16–22.

[B139] Zuo X. X. Lu H. Y. Jiang L. P. Zhang J. P. Yang X. Y. Huan X. J.. (2017). Dating rice remains through Phytolith Carbon-14 Study reveals domestication at the beginning of the Holocene. Proc. Natl. Acad. Sci. U. S. A. 114, 6486–6491. 10.1073/pnas.1704304114 28559349 PMC5488950

